# An Aromatic Sensor with Aversion to Damaged Strands Confers Versatility to DNA Repair

**DOI:** 10.1371/journal.pbio.0050079

**Published:** 2007-03-13

**Authors:** Olivier Maillard, Szilvia Solyom, Hanspeter Naegeli

**Affiliations:** Institute of Pharmacology and Toxicology, University of Zurich–Vetsuisse, Zurich, Switzerland; Scripps Research Institute, United States of America

## Abstract

It was not known how xeroderma pigmentosum group C (XPC) protein, the primary initiator of global nucleotide excision repair, achieves its outstanding substrate versatility. Here, we analyzed the molecular pathology of a unique Trp690Ser substitution, which is the only reported missense mutation in xeroderma patients mapping to the evolutionary conserved region of XPC protein. The function of this critical residue and neighboring conserved aromatics was tested by site-directed mutagenesis followed by screening for excision activity and DNA binding. This comparison demonstrated that Trp690 and Phe733 drive the preferential recruitment of XPC protein to repair substrates by mediating an exquisite affinity for single-stranded sites. Such a dual deployment of aromatic side chains is the distinctive feature of functional oligonucleotide/oligosaccharide-binding folds and, indeed, sequence homologies with replication protein A and breast cancer susceptibility 2 protein indicate that XPC displays a monomeric variant of this recurrent interaction motif. An aversion to associate with damaged oligonucleotides implies that XPC protein avoids direct contacts with base adducts. These results reveal for the first time, to our knowledge, an entirely inverted mechanism of substrate recognition that relies on the detection of single-stranded configurations in the undamaged complementary sequence of the double helix.

## Author Summary

DNA is constantly exposed to damaging agents such as ultraviolet light, carcinogens, or reactive metabolic byproducts causing thousands of DNA lesions in a typical human cell every hour. To prevent irreversible mutations, many of these different lesions are eliminated by a DNA repair system known as “nucleotide excision repair.” Repair is initiated by the XPC protein, which recognizes damaged sites in the DNA double helix. Here, we describe how the XPC protein probes the way in which the two DNA strands are aligned, and how a recurrent protein motif, termed oligonucleotide/oligosaccharide-binding fold, is used to detect dynamic fluctuations of DNA in the lesion containing regions. We show that XPC interacts preferentially with the undamaged strand opposite the lesion sites and conclude that XPC protein adopts an entirely indirect recognition mechanism to be able to detect a nearly infinite spectrum of DNA lesions.

## Abstract 3

How does XPC (an enzyme responsible for DNA repair) know where the damage is? It seems to recognize undamaged DNA displaced by the defect rather than the damaged base itself.

## Introduction

One of the most formidable challenges in DNA metabolism is that faced by the initiator of the nucleotide excision repair reaction as it locates damaged sites in the context of a large excess of mostly undamaged residues. This challenge is further complicated by an astounding diversity of target lesions, including cyclobutane pyrimidine dimers and pyrimidine–pyrimidone (6–4) photoproducts induced by UV (ultraviolet) light, bulky DNA adducts generated by electrophilic chemicals [1–4], a subset of oxidative products [5–7], and certain protein-DNA crosslinks [8]. Molecular defects in this versatile nucleotide excision repair response cause autosomal recessive disorders in humans such as xeroderma pigmentosum (XP) or Cockayne syndrome [9–11]. The XP syndrome, in particular, is characterized by photosensitivity and an extreme predisposition to sunlight-induced skin cancer [12]. In addition to cutaneous abnormalities, some XP patients also develop internal tumors [13] or neurologic complications leading to DeSanctis–Cacchione syndrome [14]. Individuals affected by XP are classified into seven repair-deficient complementation groups designated XP–A through XP–G [15].

The nucleotide excision repair response is separated in two pathways. Global genome repair (GGR) activity is responsible for the excision of DNA lesions across all nucleotide sequences, whereas transcription-coupled repair removes offending lesions only from the transcribed strand of active genes [16,17]. A principal difference between these pathways resides in the initial detection of DNA damage. During transcription-coupled repair, elongation of the RNA polymerase II complex is blocked by abnormal residues, thereby inducing the assembly of repair complexes [18]. In contrast, the GGR machinery is dependent on the initial recognition of damaged sites by XPC protein, which constitutes a universal sensor of bulky lesions [19,20]. Recent studies showed that XPC is also required for histone modifications in response to bulky lesion formation, presumably to facilitate chromatin remodeling [21,22]. It has been suggested that the recruitment of XPC protein is triggered by distortions of the DNA substrate [23–25], but how this initial factor distinguishes between normal conformations of the double helix, induced by nucleosome assembly, transcription or other physiologic processes, and the DNA deformation at damaged sites remained elusive. This lack of mechanistic knowledge reflects the fact that no structure is available for any XPC homolog. Thus, the purpose of this study was to identify a nucleic acid interaction motif that is responsible for the unique recognition function of XPC protein.

The human *XPC* gene encodes a polypeptide of 940 amino acids that exists as a complex with centrin 2, a centrosomal protein, and HR23B, one of two mammalian homologs of yeast RAD23. XPC protein itself possesses DNA-binding activity, whereas the centrin 2 and HR23B partners exert accessory functions [26,27]. Uchida et al. [28] have been able to narrow down the DNA-binding domain of XPC to a region of 137 amino acids (codons 607–742) within its evolutionary conserved carboxy-terminal half. Because most mutated *XPC* alleles in xeroderma pigmentosum families lead to premature terminations as a result of frameshifts, nonsense mutations, deletions, insertions or aberrant splicing, only one single substitution, which causes a Trp690Ser change, has been identified in the evolutionary conserved region of XPC protein [29]. Although the loss of this aromatic side chain maps to the presumed DNA-binding domain, its consequence with respect to substrate recognition in the GGR pathway is unknown, prompting a mutational screen to analyze the general role of conserved XPC residues in the detection of DNA lesions. This study disclosed an aromatic hot spot, consisting of Trp690 and Phe733, which mediates an affinity for the single-stranded character of target sites but with an astonishing aversion to associate with damaged DNA strands. A dual system of aromatics that stack with individual unpaired bases of single-stranded DNA has already been identified in RPA (replication protein A), breast cancer susceptibility 2 protein, and many other single-stranded DNA-binding factors [30–33]. Therefore, our results point to a counterintuitive mechanism of damage recognition by which XPC protein avoids direct contacts with bulky lesions but, instead, probes the local susceptibility of intact nucleotides, on the opposite side of the double helix, to adopt a single-stranded configuration. The spontaneous Trp690Ser point mutation associated with the XP syndrome interferes with this inverted mode of substrate discrimination.

## Results

### Identification of Evolutionary Conserved Aromatic Residues

**Figure 1:**
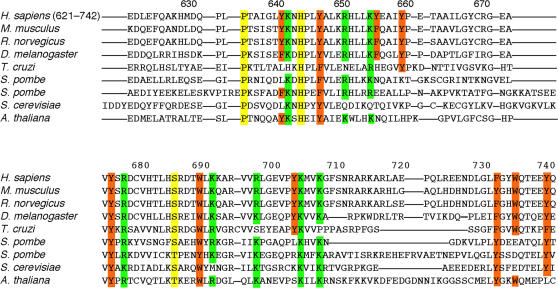
Evolutionary Conserved Residues in the Proposed DNA-Binding Domain of XPC Protein Sequence comparison between eukaryotic XPC homologs. There are two homologous genes in S. pombe. Amino acids targeted by site-directed mutagenesis are highlighted. Y, F, W, aromatic (orange); K, L, positively charged (green); P, H, S, other highly conserved positions (yellow).

The human XPC sequence has been aligned [34] with its homologs from mouse, rat, Drosophila melanogaster, Trypanosoma cruzi, yeast, and Arabidopsis thaliana to identify potential consensus motifs in a region that includes the presumed DNA-binding domain [28]. This sequence alignment demonstrates that Trp690, mutated in an XP family, is maintained from lower eukaryotes to plants and mammals. The only exception is provided by one of the two homologs in Schizosaccharomyces pombe, where the regular Trp at this position is replaced by another aromatic residue (Figure 1). The molecular function of an obligatory aromatic side chain at codon 690 was tested by a systematic comparison with all other evolutionary conserved aromatics that were identified in the same portion of human XPC protein, i.e., between codons 531 and 742. Also, the effects of these mutations were evaluated in relation to the substitution of other conserved residues with varying side chains. Figure 1 shows the positions in the presumed DNA-binding domain that have been selected for site-directed mutagenesis and highlights their degree of conservation among eukaryotes.

### Conserved Aromatics Are Critical Determinants of XPC Function

A host cell reactivation assay was used to monitor the DNA repair proficiency of XPC mutants in human cells [35]. XP–C fibroblasts, which fail to express XPC protein, were transiently transfected with a dual luciferase reporter system accompanied by an expression vector coding for human XPC protein or the different mutants. The reporter construct, which carries a firefly luciferase gene, was damaged by exposure to UV light (254 nm; 1000 J/m^2^) and supplemented with an unirradiated control vector that expresses the *Renilla* luciferase. Following varying repair times, firefly luciferase activity was determined in cell lysates and normalized against the internal *Renilla* standard.

**Figure 2:**
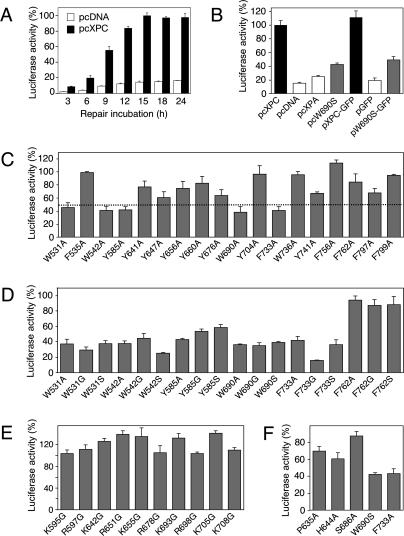
Screening for Repair-Deficient XPC Mutants (A) Time course of host cell reactivation assay. Human XP–C fibroblasts were transfected with an expression vector coding for wild-type XPC (pcXPC) or the empty control vector (pcDNA). Repair complementation was assessed after the indicated times by monitoring luciferase expression from a UV-irradiated reporter construct. Excision is reported as the percentage of wild-type activity after 15 h of incubation (± SD). (B) Specificity of the repair assay. XP–C fibroblasts were transfected with expression vectors coding for wild-type XPC (pcXPC or pXPC–GFP), wild-type XPA (pcXPA), the control vectors (pcDNA and pGFP), or vectors containing the Trp690Ser mutant sequences (pcW690S or pW690S–GFP). Excision is reported as the percentage of wild-type activity (15-h incubations). (C) Deletion of aromatic side chains. XP–C fibroblasts were transfected with vector pcXPC carrying the indicated mutations. DNA repair is expressed as the percentage of wild-type complementation (15-h incubations) after deduction of background luciferase activity obtained with the control vector. The dashed line indicates a threshold of 50% reduction in repair activity. (D) Replacement of aromatic residues by amino acids of different properties. (E) Deletion of the positively charged side chains of conserved Lys or Arg residues. (F) Ala substitutions of absolutely conserved positions in the center of the DNA-binding domain.

Due to the repair defect of XP–C cells, transcription of the reporter gene was suppressed by persistent UV lesions, resulting in reduced firefly luciferase activity. However, DNA repair and, hence, firefly luciferase expression was restored following transfection with pcXPC, demonstrating that the genetic defect of XP–C fibroblasts is corrected by wild-type XPC protein (Figure 2A). In contrast, expression of the reporter gene was not rescued when the same XP–C cells were transfected with the empty vector pcDNA (Figure 2B). The residual background activity (∼15% of wild-type control), observed in the presence of these empty vectors, is likely due to the transcription-coupled repair process, which operates independently of XPC. In part, this residual activity may also result from a minor fraction of plasmids remaining free of bulky UV lesions in the luciferase reporter sequence.

The firefly luciferase production was not restored when, instead of XPC, XPA protein was expressed in XP–C fibroblasts (Figure 2B), thus demonstrating the specificity of our host cell reactivation system. Also, the firefly luciferase production was inhibited when the XPC sequence was modified to carry the Trp690Ser mutation responsible for clinical manifestations of the XP syndrome (Figure 2B). Nearly identical results were obtained by transfecting the cells with vector pXPC–GFP, which drives the expression of wild type or mutated XPC sequences fused, on their carboxy-terminal side, to green fluorescent protein (GFP). As expected, no complementation of the repair defect was detected upon expression of GFP alone using the corresponding control vector (Figure 2B).

The relative luciferase activity indicative of DNA repair was determined in the presence of each site-directed mutant, and the results were reported as the percentage of wild-type complementation after deduction of background luciferase expression. Initially, the aromatic side chains of conserved Phe, Trp, and Tyr residues were eliminated by Ala substitutions (Figure 2C). In most cases, the excision-repair proficiency of XPC protein was only marginally diminished by these Phe→Ala, Trp→Ala, or Tyr→Ala changes. However, point mutations at the conserved codons 531, 542, 585, 690, and 733 resulted in a substantial (>50%) reduction of excision activity, and the residual DNA repair observed with these mutants is similar to the low level of complementation promoted by the Trp690Ser allele (Figure 2C). All these mutants displayed essentially the same repair deficiency when reexamined as GFP fusion products (unpublished data).

The more sensitive codons 531, 542, 585, 690, and 733 were further tested by converting the respective aromatics to different amino acids with varying properties. In all cases, the luciferase activity reflecting DNA excision repair was strongly reduced regardless of whether the aromatics were replaced by the aliphatic side chain of Ala, the hydrogen moiety of Gly, or the hydrophilic side chain of Ser (Figure 2D). These results imply that the loss of activity conferred by these XPC mutations is primarily a consequence of the missing aromatic residue rather than being dependent on the properties of the newly introduced substituent.

Basic amino acids frequently make contacts with the phosphate moieties of the DNA backbone. Thus, evolutionary conserved Lys and Arg residues, located between codons 595 and 708 of the human XPC protein, were targeted by site-directed mutagenesis. The positively charged side chains were eliminated by changing the respective residues to Gly, but none of the resulting Lys→Gly or Arg→Gly substitutions were able to perturb the XPC function (Figure 2E). In addition, absolutely conserved amino acids in the center of the putative DNA-binding domain of human XPC protein were changed to Ala residues. The resulting Pro635Ala, His644Ala, and Ser686Ala substitutions reduced the luciferase activity to a moderate degree but, interestingly, none of these mutants reached the low residual repair level observed after removal of an aromatic side chain at position 690 or 733 (Figure 2F).

### Normal Expression and Cellular Localization of Repair-Deficient XPC Mutants

**Figure 3:**
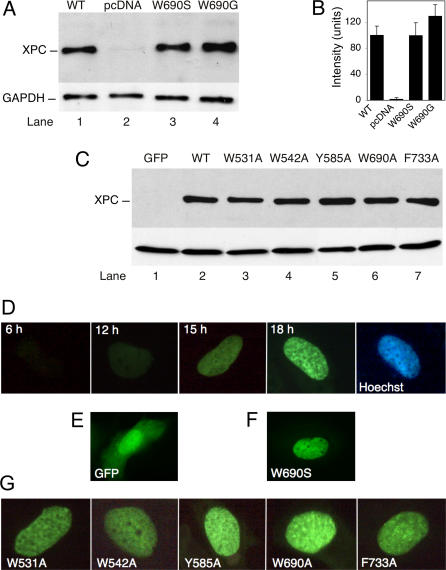
Normal Cellular Expression and Localization of Repair-Deficient XPC Mutants (A) Immunoblot analysis of XP–C fibroblasts transfected with pcXPC vectors coding for wild-type protein or repair-deficient mutants. Soluble cell lysates (20 μg) were separated on polyacrylamide gels and probed for XPC protein using a specific monoclonal antibody directed against the C-terminal sequence of human XPC. Glyceraldehyde-3-phosphate dehydrogenase (GAPDH) was recorded as the internal standard. Lane 2: XP–C fibroblasts transfected with the pcDNA control vector. (B) Densitometric quantification of three to five independent experiments. The intensity of immunoreactive bands corresponding to XPC protein was normalized against the glyceraldehyde-3-phosphate dehydrogenase standard and reported as the percentage of the wild-type signal (± SD). (C) Immunoblot analysis of XP–C fibroblasts transfected with pGFP (lane 1), or the vectors coding for wild-type (lane 2) or mutant XPC proteins (lanes 3–7) fused to GFP. The primary antibody was directed against GFP. (D) Time course of fluorescent fusion protein expression in XP–C fibroblasts transfected with pXPC–GFP containing the wild-type sequence. Nuclei were stained with Hoechst reagent. (E) Distribution of GFP in XP–C fibroblasts. (F and G) Representative images demonstrating the nuclear localization of repair-deficient mutants 15 h after transfection.

The cellular XPC content was monitored by immunoblot analysis of XP–C fibroblasts harvested 15 h after transient transfections with vector pcXPC, promoting the expression of human XPC alone, or vector pXPC–EGFP translating to the production of XPC as a GFP fusion protein. In both cases, a quantitative comparison of protein levels demonstrated that the Trp690Ser and Trp690Gly mutants were expressed in human fibroblasts to similar levels as the wild-type counterpart (Figure 3A and 3B). Moreover, the repair-deficient mutants with Ala substitutions at codons 531, 542, 585, 690, and 733 were detected in human fibroblasts in nearly identical amounts as wild-type XPC protein (Figure 3C). Thus, the repair deficiency observed by substituting these conserved aromatics is not a consequence of reduced XPC expression or enhanced degradation.

The GFP fusion partner was exploited to perform fluorescence microscopy studies. A time course experiment with the wild-type sequence demonstrated that expression of the XPC–GFP fusion increases during incubation periods of 18 h after transfection, with a cellular localization that is predominantly restricted to the nucleus (Figure 3D). Control cells transfected with vector pGFP demonstrated that GFP alone displays a more diffuse distribution extending to both the cytoplasma and nucleus (Figure 3E). However, the strong nuclear localization is reestablished after expression of GFP fused to the Trp690Ser mutant (Figure 3F). A similar level of fluorescence with the same characteristic nuclear localization was recorded for each of the repair-defective Ala mutants (Figure 3G). These results demonstrate that the repair deficiency of these tested mutants is not due to defective translocation into the nuclear compartment.

### XPC Protein Displays a Single-Stranded DNA-Binding Motif

**Figure 4:**
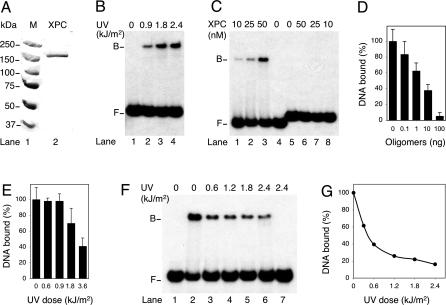
Affinity of XPC Protein for Native Single-Stranded Oligonucleotides (A) Analysis of MBP–XPC fusion protein by Coomassie staining of a denaturing 8% polyacrylamide gel. Lane 1, markers; lane 2, purified fraction. (B) Electrophoretic mobility shift assay demonstrating the preference of wild-type XPC protein for UV-irradiated duplexes over the unirradiated control (lane 1). Radiolabeled double-stranded DNA fragments of 65 base pairs (2 nM) were incubated for 30 min with the MBP–XPC fusion product (50 nM) and duplex poly[dI-dC] (10 ng/μl). F, free DNA; B, protein-bound DNA. (C) Preference of XPC protein for binding to single-stranded 65-mer oligonucleotides (lanes 1–4) relative to undamaged 65-mer duplexes (lanes 5–8). (D) Competition with single-stranded DNA. Radiolabeled 65-mer duplexes were UV-irradiated (1.8 kJ/m^2^) and incubated at a concentration of 2 nM with XPC protein (50 nM), increasing amounts of unlabeled single-stranded oligomers of 65 nucleotides, and duplex poly[dI-dC] (10 ng/μl). The fractions of protein-bound oligomers were determined by electrophoretic mobility shift assay, quantified by laser scanning densitometry, and expressed as the percentage of binding observed in the absence of competitor DNA (± SD). (E) Competition with double-stranded DNA. Radiolabeled 65-mer oligonucleotides (2 nM) were incubated with XPC protein (50 nM), 100 ng of plasmid DNA (pcDNA) exposed to the indicated UV doses, and duplex poly[dI-dC] (10 ng/μl). The fractions of protein-bound oligomers were quantified and expressed as the percentage of binding determined in the presence of undamaged competitor DNA (± SD). (F) Suppression of single-stranded DNA binding by UV irradiation. Radiolabeled 65-mer oligonucleotides (2 nM), exposed to the indicated UV doses, were incubated for 30 min with XPC protein (100 nM) and duplex poly[dI-dC] (10 ng/μl). Lane 1: no XPC protein. (G) Quantification by laser scanning densitometry of two independent experiments performed with UV-irradiated single-stranded oligonucleotides (± SD).

The wild-type XPC polypeptide was coupled to maltose-binding protein (MBP), produced in Spodoptera frugiperda (*Sf*9) cells and purified to homogeneity by nickel and heparin affinity chromatography. MBP was chosen as a fusion partner to promote solubility and proper folding [36]. Another advantage of the MBP tag is that, on its own, it lacks DNA-binding activity [37]. On sodium dodecylsulfate gels, the final fraction of the MBP–XPC fusion product migrated as a single band with an apparent molecular weight of ∼170 kDa, which corresponds to the expected size of the 125-kDa XPC protein linked to the 43-kDa MBP moiety (Figure 4A).

Conflicting results regarding the affinity of XPC protein for DNA substrates of different lengths and conformations have emerged. Oligonucleotides with fewer than 60 base pairs resulted in weakened binding and reduced damage selectivity [25,38,39]. As a consequence, we employed radiolabeled duplexes of 65 base pairs to monitor DNA binding in electrophoretic mobility shift assays. The nucleotide sequence was designed to contain neighboring pyrimidines for the formation of UV-induced dimers. Thus, the double-stranded substrates were UV irradiated (254-nm wavelength) to test the DNA damage selectivity of purified XPC fusion products. As expected from previous reports [40,41], an increased affinity of XPC for UV-irradiated duplexes, over the unirradiated control, was detected when the binding reactions were supplemented with an excess of undamaged competitor DNA, i.e., under conditions of limiting protein (Figure 4B).

In addition to this known affinity for UV-irradiated duplexes, we observed that XPC protein exhibits an extraordinary preference for binding to single-stranded 65-mer oligonucleotides over undamaged double-stranded fragments of the same length (Figure 4C). These results obtained with relatively long oligomeric substrates imply that the XPC subunit fits the classic definition of a single-stranded DNA-binding protein. Because shorter duplexes are more prone to spontaneous denaturation, generating regions of single-stranded DNA, the preference of XPC protein for binding to single strands over double-stranded DNA is abrogated by reducing the oligonucleotide length to 40 residues or fewer (unpublished data). This effect of substrate length provides a possible explanation for the diverging results of previous studies where the damage selectivity of XPC protein had not been attributed to an affinity for single-stranded DNA conformations [23,40]. A striking bias for single-stranded DNA is further supported by competition assays showing that the binding of XPC protein to UV-irradiated 65-mer duplexes is sensitive to the addition of 65-mer single strands (Figure 4D). Conversely, when the competitor consisted of double-stranded plasmids, an excess of heavily UV-irradiated DNA was necessary to reduce the binding of XPC protein to single-stranded oligonucleotides (Figure 4E).

Subsequently, we observed that the high-affinity association of XPC protein with DNA single strands was progressively reduced upon UV irradiation of the oligonucleotide substrate (Figure 4F). Interestingly, the UV dose of 600 J/m^2^ is expected to yield a damage frequency of <1 photoproduct/oligonucleotide molecule (40), yet this low level of radiation was sufficient to reduce the single-stranded DNA-binding activity of XPC protein by ∼50%. Higher UV doses further suppressed the single-stranded DNA-binding activity to marginal levels (Figure 4G), indicating that bulky lesions collide with the ability of XPC protein to form complexes with DNA oligonucleotides. Taken together, we conclude that XPC protein is recruited to target sites by virtue of its characteristic preference for deoxyribonucleotide sequences that adopt a single-stranded conformation. Surprisingly, this sensor protein associates preferentially with undamaged strands but rejects direct interactions with damaged strands.

### The Trp690Ser Substitution Confers Defective DNA Binding

**Figure 5:**
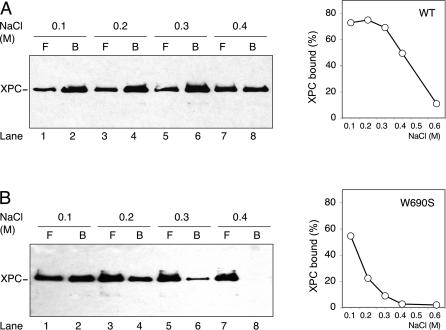
The Trp690Ser Mutant Is Defective in Substrate Binding (A) Pull-down assays were performed by coincubating *Sf*9 cell lysate (5 μl) containing wild-type XPC protein and 50 μl of single-stranded DNA beads. The binding buffer was supplemented with the indicated concentrations of NaCl. The fractions of free (F) and bound (B) protein were separated and analyzed by gel electrophoresis and immunobloting using specific monoclonal antibodies. The panel on the right provides a quantitative evaluation of three independent binding assays showing the proportion of pulled-down XPC protein at the different ionic strengths. (B) Pull-down assay with *Sf*9 cell lysate (5 μl) containing the Trp690Ser mutant (left) and quantitative evaluation of three independent experiments (right).

Two different strategies were used to test the ability of XPC mutants to interact with single-stranded DNA substrates. First, MBP–XPC fusion products were expressed in *Sf*9 cells, and the respective cell lysates were incubated with single-stranded DNA immobilized on agarose beads. After 2 h-incubations at 4 °C, the fraction of XPC protein in the pellet (bound to DNA) was separated by repeated washing from the free XPC molecules remaining in the supernatant. The extensively washed pellets and the accompanying supernatants were analyzed separately by gel electrophoresis and immunoblotting. Side-by-side comparisons showed that, in the case of the wild-type control, a major proportion (>70%) of XPC protein was recovered in the DNA-agarose pellet when the binding reactions were performed in buffer containing NaCl concentrations of 0.1–0.3 M (Figure 5A, lanes 1–6). If the NaCl concentration was raised to 0.4 M, only ∼50% of wild-type protein remained bound to DNA (Figure 5A, lanes 7 and 8). When the ionic strength was further increased, the proportion of XPC protein retained in the DNA pellet was diminished, reflecting a gradual reduction of nucleic acid binding. In the case of the Trp690Ser mutant, the fraction of protein recovered in association with the DNA beads was markedly reduced already in buffer containing 0.1 M NaCl (Figure 5B, lanes 1 and 2). When the NaCl concentration was increased to 0.2 or 0.3 M, the proportion of mutant XPC protein remaining in the DNA pellets was further reduced to ∼20% or less (Figure 5B, lanes 3–6). Essentially none of the Trp690Ser mutant remained assembled with DNA when the NaCl concentration was raised to 0.4 M (Figure 5B, lanes 7 and 8). These results show that the Trp690Ser substitution identified in an XP family disrupts the affinity of XPC protein for its DNA substrate.

### Trp690 and Phe733 Define an Aromatic Hotspot for Substrate Recognition

**Figure 6:**
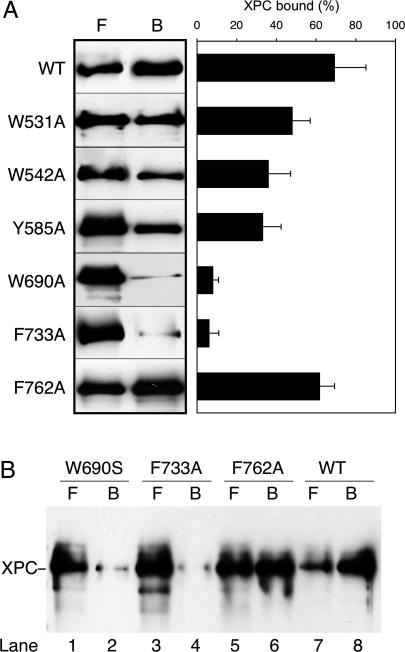
DNA-Binding Deficiency of Trp690 and Phe733 Mutants (A) Pull-down assays were performed by coincubating *Sf*9 cell lysate (5 μl) containing wild-type XPC or the indicated Ala mutants and single-stranded DNA beads. The binding buffer contained 0.3 M NaCl. The fractions of free (F) and bound (B) protein were separated and analyzed by gel electrophoresis and immunobloting using specific monoclonal antibodies. The panel on the right shows the quantitative evaluation of three independent binding assays (± SD). (B) Side-by-side comparison of the DNA-binding capacity of wild-type XPC protein (lanes 7 and 8) and the indicated mutants (lanes 1–6).

All repair-deficient substitutions were expressed as MBP fusion products and tested for their ability to interact with single-stranded DNA immobilized on agarose beads. This systematic comparison was performed in buffer containing 0.3 M NaCl, which corresponds to the ionic strength under which the most pronounced difference was detected between wild-type XPC protein and the Trp690Ser reference. Under these conditions, the three mutants Trp531Ala, Trp542Ala, and Tyr585Ala, which carry Ala substitutions outside the presumed DNA-binding domain, displayed a gradually reduced DNA-binding capacity compared to wild-type XPC protein (Figure 6A), possibly reflecting indirect structural effects on the substrate recognition surface. This gradient of decreasing interactions with DNA culminated in the nearly complete loss of substrate binding in response to the Trp690Ala or Phe733Ala substitution. In both cases, the vast majority of mutant Trp690Ala and Phe733Ala protein appeared as free molecules in the supernatant, and only an insignificant fraction of these two species remained bound to the single-stranded DNA agarose beads (Figure 6A). The Phe762Ala substitution, which yielded only a mild DNA repair defect in the host cell reactivation assay, was included in this nucleic acid-binding screen as an additional control. In full agreement with its in vivo repair proficiency, this Phe762Ala mutant was able to associate with the DNA substrate nearly as efficiently as the wild-type counterpart.

Among the repair-deficient XPC mutants identified in this study, only the Phe733Ala substitution resulted in the same poor DNA-binding activity as the XP mutation at codon 690. Therefore, an independent preparation of this Phe733Ala mutant (Figure 6B, lanes 3 and 4) was reexamined for DNA binding in comparison with newly prepared cell lysates containing the repair-deficient Trp690Ser mutant (lanes 1 and 2), the repair-proficient Phe762Ala derivative, (lanes 5 and 6) as well as the wild-type XPC control (lanes 7 and 8). This control experiment, again carried out in the presence of 0.3 M NaCl, confirmed that the removal of an aromatic side chain at positions 690 and 733 disrupts the DNA-binding function of XPC protein. Thus, the molecular defect underlying the prominent repair deficiency of these Trp690 and Phe733 substitutions resides with the inability of the respective mutants to undergo close contacts with the DNA substrate.

### Probing of XPC Mutants with Single-Stranded Oligonucleotides

**Figure 7:**
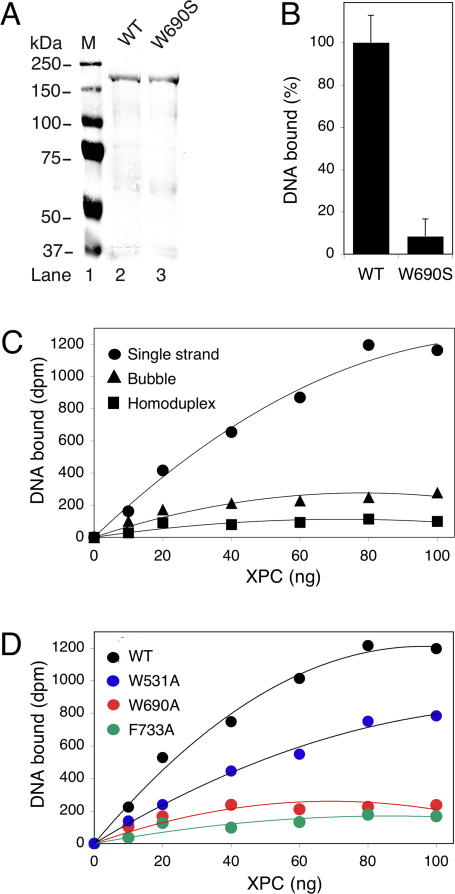
Single-Stranded Oligonucleotide-Binding Defect (A) Analysis of immunoprecipitated XPC by Coomassie staining of a denaturing 8% polyacrylamide gel. The MBP–XPC fusions were purified from *Sf*9 lysates using monoclonal anti-MBP antibodies linked to paramagnetic beads. Lane 1, markers; lane 2, wild-type MBP–XPC; lane 3, fusion protein containing the Trp690Ser reference mutation. (B) Binding of wild-type XPC and Trp690Ser mutant to single-stranded oligonucleotides. Immunoprecipitated MBP–XPC protein (100 ng, 3 nM) was incubated with ^32^P-labeled 65-mer oligonucleotides (2 nM). The DNA molecules captured by XPC protein were separated from the free oligonucleotides and quantified in a scintillation counter. Single-stranded DNA-binding activity (± SD) is reported as the radioactivity immobilized by XPC after deduction of the background binding determined with empty beads. (C) Differential binding to distinct DNA conformations. Immunoprecipitated MBP–XPC protein (100 ng, 3 nM) was incubated with ^32^P-labeled substrates (2 nM) consisting of 65-mer homoduplexes, 65-mer heteroduplexes with a central 3-nucleotide bubble, or 65-mer single-stranded oligonucleotides. The DNA molecules captured by XPC protein were separated from free DNA and quantified in a scintillation counter. DNA-binding activity (mean values of two experiments) is reported as the radioactivity immobilized by XPC after deduction of the background binding determined with empty beads. (D) Comparison between wild-type XPC and Ala mutants. Paramagnetic beads containing the indicated amounts of immunopurified MBP–XPC protein were incubated with ^32^P-labeled 65-mer oligonucleotides (2 nM). DNA associated with XPC protein was separated from the free oligonucleotides and quantified in a scintillation counter. Single-stranded DNA binding activity (mean values of four experiments) is reported as the radioactivity immobilized by XPC after deduction of the background binding to empty beads.

A second experimental strategy, based on defined oligonucleotide probes, was established to confirm that the mutations at codons 690 and 733 confer defective binding to single-stranded DNA. For that purpose, MBP–XPC products were first purified from *Sf*9 cell lysates by immunoprecipitation with anti-MBP antibodies linked to paramagnetic beads. This one-step procedure generated nearly homogenous preparations of MBP–XPC fusion proteins (Figure 7A). Subsequently, the amount of paramagnetic beads was adjusted to include 100 ng of purified protein, translating to a final XPC concentration of 3 nM in each binding reaction. Such purified fractions of wild-type protein or Trp690Ser mutant were incubated with radiolabeled 65-mer single strands and, following 2 h at 4 °C, the oligonucleotides captured by XPC protein were separated from free DNA. After extensive washing, the radioactivity associated with XPC protein on the paramagnetic beads was quantified by scintillation counting. We found substantial binding of wild-type XPC protein to single-stranded oligonucleotides but this interaction was markedly reduced when the Trp690Ser mutant was tested under exactly the same conditions (Figure 7B). Next, the reaction mixtures were adjusted to contain different amounts of protein, thus demonstrating a dose-dependent increase of DNA-binding activity in the presence of wild-type XPC. These dose-dependence experiments confirmed that XPC protein interacts more efficiently with 65-mer heteroduplexes containing a 3-nucleotide bubble than to perfectly homoduplex controls (Figure 7C). The DNA-binding activity was further enhanced by replacing duplex substrates with single-stranded oligonucleotides of the same length (Figure 7C). Finally, these dose-dependent binding assays were used to compare the relative affinity of wild-type and mutant proteins for single-stranded DNA. In contrast to the efficient association of wild-type XPC with 65-mer oligonucleotides, the ability to interact with single-stranded DNA was essentially lost when we tested the mutants carrying an Ala substitution at codon 690 or 733 (Figure 7D). However, in agreement with the different assay of Figure 6, the DNA-binding activity was more moderately affected by a Trp531Ala substitution (Figure 7D). These results support the conclusion that the two aromatic residues Trp690 and Phe733 are critically required for the recognition of single-stranded DNA conformations.

## Discussion

The most astounding feature of the GGR machinery is its ability to eliminate a wide diversity of DNA lesions, but how this repair system discriminates anomalous residues against the vast background of normal deoxyribonucleotides is still a focus of intense research, mainly because there is no common chemical motif among the different DNA adducts that would account for a classic “lock and key” recognition scheme [1–4]. Our mutagenesis screen designed to probe the mode of action of human XPC protein indicates that this primary initiator of the GGR reaction donates a pair of aromatic side chains (Trp690 and Phe733) to monitor the double helical integrity of DNA and to recognize the local single-stranded character imposed on the undamaged side of the DNA duplex. These novel findings have several important implications with regard to damage recognition and the versatile GGR pathway.

First, the preference of XPC protein for substrates containing a short single-stranded segment, over fully complementary duplexes, provides a truly universal mechanism for the detection of lesion sites. Normally, the native DNA duplex is stabilized by complementary base pairing as well as by stacking interactions between adjacent bases such that, in the absence of damage, the bases are positioned to the interior of the double helix. In contrast, DNA at damaged sites deviates considerably from this canonical Watson–Crick geometry. Bulky adducts often disrupt normal pairing and stacking interactions, thereby lowering the thermal and thermodynamic stability of the duplex, which results in local separation of the complementary strands and exposure of unpaired and unstacked bases on the surface of the double helix, thus generating an abnormal configuration with features that resemble single-stranded DNA [24]. The present equilibrium binding studies as well as kinetic measurements [25], both demonstrating an extraordinary affinity for single-stranded oligonucleotides relative to double-stranded counterparts, imply that only base adducts that destabilize the double helix generate the key molecular signal for recognition by the single-stranded DNA-binding motif of XPC protein.

**Figure 8:**
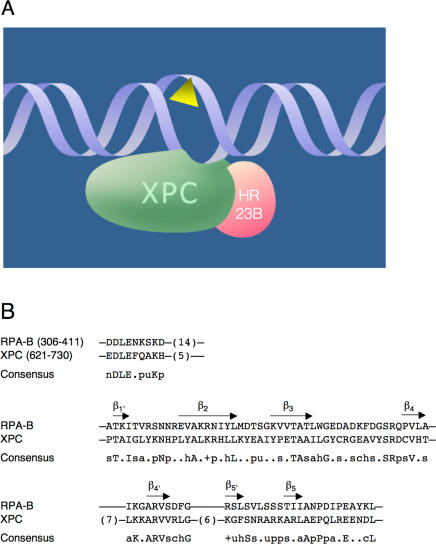
Versatile Damage Recognition: Detection of Single-Stranded Configurations in the Undamaged Strand of the Double Helix (A) Initial interaction of XPC protein with damaged sites driven by an affinity for native single-stranded DNA. The triangle symbolizes a helix-distorting bulky lesion. This mechanism with inverted DNA strand specificity directs XPC protein to the undamaged strand and the downstream factors of the GGR pathway to the damaged strand. (B) Alignment of the RPA-B and XPC DNA-binding sequences. The consensus was derived using the following amino acid classes [47]: hydrophobic (h, ALICVMYFW); the aliphatic subset of these (a, ALIVMC); small (s, ACDGNPSTV); the “tiny” subset of these (u, GAS); polar (p, CDEHKNQRST); charged (c, DEHKR), positively charged (+, HKR); and negatively charged (n, DE). The length of nonalignable gaps is indicated in parentheses and the β-sheet elements are indicated by the arrows.

Second, our results point to an inverted mode of recruitment mediated by an affinity for the undamaged strand of the DNA duplex. In fact, we observed an unfavorable binding of XPC protein to UV-irradiated DNA oligonucleotides compared to undamaged single-stranded counterparts. A similar reduction of oligonucleotide binding has been detected following the introduction of a site-specific cisplatin adduct [25], implying that the interaction of XPC protein with single-stranded DNA is generally disturbed by the presence of adducted, crosslinked, or otherwise aberrant base residues. Thus, the exquisite affinity of XPC protein for single-stranded oligonucleotides, in combination with its aversion to interact with damaged strands, indicates that the recognition step in the GGR pathway is guided by an initial association with the native strand of damaged duplexes (Figure 8A), without ruling out the possibility that XPC protein may ultimately interact with both strands. Such an inverted mode of damage recognition, which is completely independent of the variable chemistry of the lesion sites, accommodates the ability of the GGR machinery to detect a very wide array of DNA adducts. Recently, it has been reported that RPA is equally refractory to interactions with damaged oligonucleotides [42], suggesting a functional analogy between XPC protein and representatives of the large family of single-stranded DNA-binding factors.

Third, the dependence on a dual system of aromatic amino acids indicates a structural basis for the observed similarity between the XPC subunit and known single-stranded DNA-binding proteins. We found that two distinct aromatics in the presumed nucleic acid-binding domain of XPC protein, i.e., Trp690 and Phe733, are more critically involved in the high-affinity interaction with single-stranded configurations than all other conserved residues in the same XPC region. Even mutations affecting the absolutely conserved Pro635, Lys642, His644, Tyr676, Arg678, Ser686, or Lys708, located in the DNA-binding domain, cause less incisive repair deficiencies than the removal of the aromatic side chains at positions 690 and 733. Other aromatic side chains at codons 531, 542, and 585 are similarly required for excision repair activity, but their removal confers more moderate DNA-binding defects. This observation is consistent with a previous report indicating that residues 531–585 are located outside the core DNA-binding domain [28]. The distinctive requirement for a pair of aromatics (Trp690 and Phe733 in the case of XPC) is reminiscent of the OB-fold of many single-stranded DNA-binding proteins [30]. In RPA, for example, four different DNA-binding subdomains with the characteristic OB-fold are responsible for the association with single-stranded substrates [33]. Each of these domains forms a small β-barrel consisting of several short elements of secondary structure connected by loops of variable length [43]. The single-stranded DNA-binding activity of these RPA subdomains correlates with the presence of two structurally conserved aromatics that mediate stacking interactions with closely spaced DNA bases. Other OB-folds in the RPA complex that lack these aromatic side chains fail to contribute to nucleic acid binding [33]. The reiteration of a pattern of two separate aromatics in the DNA-binding domain of XPC protein lends support to the hypothesis that this repair factor may display an analogous structural fold to recognize DNA bases extruded from the double helix, and forced into a single-stranded conformation, as a consequence of bulky lesion formation. The different OB-fold subdomains of RPA range between 110 and 180 amino acids in length. As a minimal DNA-binding fragment of XPC protein has been mapped to a region of 136 amino acids [28], we predict that XPC displays a monomeric variant of this motif to detect the single-stranded character resulting from separation of just one or, depending on the extent of DNA distortion, no more than a few base pairs at lesion sites.

To summarize, XPC protein displays a range of properties that are typical of the OB-fold of single-stranded DNA-binding factors, i.e., an affinity for single-stranded oligonucleotides, an exquisite preference for undamaged strands relative to damaged strands, the pairwise deployment of aromatics for nucleic acid binding, and the ability to interact with single-stranded DNA under conditions of elevated ionic strength. This combination of functional and structural analogies raises the question of whether a common sequence motif may be shared by XPC and known single-stranded DNA-binding proteins. A systematic analysis of the XPC full-length sequence did not reveal any signature that may have predicted its DNA-binding properties [44,45]. However, a homology search focused on the comparison with the growing family of OB-fold proteins showed that the nucleic acid-binding region of XPC protein displays a remarkable similarity to one of the oligonucleotide-binding subdomains of human RPA (Figure 8B). This comparison yielded 27% identity and 73% similarity between the DNA-binding domain of XPC protein and the RPA-B motif situated in the large subunit of the human RPA complex. The sequence homology extends over most of the conserved elements of secondary structure of the RPA-B subdomain and exceeds the 12% identity detected when known OB-folds were aligned according to their high-resolution structure [32]. The same DNA-binding region of XPC also displays a 66% similarity with the OB1 and a 64% similarity with the OB2 motif of breast cancer susceptibility 2 (unpublished data). Thus, the aromatic sensor domain of XPC protein, responsible for the recognition of DNA damage in the GGR pathway, is related to the OB-folds of known single-stranded DNA-binding proteins.

In conclusion, this article shows that a versatile sensor of DNA damage achieves its wide recognition function by avoiding direct contacts with injured residues. Instead, XPC protein exploits the inherent redundancy of the genetic code in the DNA double helix to detect DNA damage in an indirect but highly versatile manner. If one strand contains a bulky lesion, normal base pairing and stacking interactions are compromised, and the intact complementary strand converts to a local single-stranded configuration, thus generating the universal molecular signal for XPC recruitment.

## Materials and Methods

### Site-directed mutagenesis.

The human *XPC* complementary DNA [38] was cloned into pcDNA3.1 (Invitrogen, http://www.invitrogen.com) using the restriction enzymes *Not*I and *Kpn*I and into pEGFP-N3 (Clontech, http://www.clontech.com) using the *Kpn*I and *Xma*I sites. Mutagenesis was carried out with the QuickChange site-directed mutagenesis kit (Stratagene, http://www.stratagene.com) following the manufacturer's instructions. Forward and reverse primers are listed in Table S1. The resulting clones were sequenced (Microsynth, http://www.microsynth.ch) to exclude accidental mutations introduced elsewhere in the complementary DNA.

### Host cell reactivation assay.

Simian virus 40-transformed human XP–C fibroblasts (GM16093) were from the Coriell Cell Repository (http://ccr.coriell.org). These cells were grown in Dulbecco's modified Eagle's medium (Gibco, http://www.invitrogen.com), supplemented with 10% fetal bovine serum, penicillin G (100 units/ml) and streptomycin (100 μg/ml), in a 5% CO_2_ humidified incubator. The pGL3 and phRL–TK vectors expressing firefly (*Photinus*) and *Renilla* luciferase, respectively, were from Promega. DNA was UV-irradiated at a concentration of 1 mg/ml in 10 mM Tris-HCl, (pH 8), and 1 mM EDTA. XP–C cells were transfected in a 6-well plate at a confluence of 95% using Lipofectamine Plus reagent (Invitrogen). Each transfection mixture contained 0.23 μg pGL3 (UV-irradiated), 0.02 μg phRL–TK (unirradiated), and 0.25 μg of the appropriate expression vector. After a 4-h incubation, the transfection reagents were replaced by complete medium. Unless otherwise indicated, the cells were lysed after another 15-h period to measure firefly and *Renilla* luciferase activity using the Dual–Luciferase assay system (Promega, http://www.promega.com) on a microtiter plate luminometer (Dynex, http://www.dynextechnologies.com). All results (mean values of at least five determinations) were normalized by calculating the ratios between firefly and *Renilla* luciferase activity. Expression of XPC polypeptides in human cells was monitored by Western blotting (using monoclonal antibodies against GFP from Clontech) and fluorescence microscopy as described [46].

### Expression and purification of XPC protein.

A polyhistidine-MBP–XPC fusion product was constructed by inserting a 2.9-kb fragment, which contains the human *XPC* complementary DNA, into the pFastBac HTc vector (Invitrogen) using the *Not*I and *Kpn*I restriction sites. Subsequently, a 1.2-kb fragment containing the *MalE* complementary DNA (from pMal-c2; New England Biolabs, http://www.neb.com) was inserted on the 5′ side of the *XPC* sequence using the *Stu*I restriction site. Recombinant baculovirus for the infection of *Sf*9 cells was generated using the BAC-TO-BAC Baculovirus Expression System (Invitrogen) following the manufacturer's instructions.

Polyhistidine- and MBP-tagged XPC protein was fractionated from *Sf*9 cell lysates [38] with two chromatographic cycles through a Ni^2+^ column (Qiagen, http://www.qiagen.com). The fractions were analyzed using a mouse monoclonal antibody against recombinant human XPC protein (Abcam, http://www.abcam.com). Samples containing XPC protein, eluting mainly at 100 mM imidazole, were pooled, dialyzed against phosphate buffer (25 mM sodium phosphate, [pH 7.8], 10% [v/v] glycerol, 5 mM β-mercaptoethanol, and 0.25 mM phenylmethane sulfonyl fluoride) containing 0.2 M NaCl, and further processed by heparin chromatography (Amersham, http://www.amershambiosciences.com). The heparin column was eluted with a 0.2–1 M gradient of NaCl. The samples containing homogeneous MBP–XPC protein, eluting at 600 mM NaCl, were pooled, dialyzed, and supplemented with glycerol to a concentration of 25% (v/v) before freezing at −80 °C. Protein concentration was determined using the Bio-Rad protein assay reagent (http://www.bio-rad.com).

A one-step purification was performed by mixing crude *Sf*9 cell lysates (5–20 μl) with monoclonal antibodies against MBP that were covalently linked to paramagnetic beads (New England BioLabs). The binding buffer consisted of 25 mM Tris-HCl, (pH 7.5), 10% glycerol, 0.01% Triton X-100, 0.25 mM phenylmethane sulfonyl fluoride, 1 mM EDTA, and 0.3 M NaCl. After incubation at 4 °C for 2 h, the beads were washed four times, and bound proteins were analyzed by denaturing gel electrophoresis followed by Coomassie staining. The yield of MBP–XPC protein was determined by quantitative laser densitometry of the 170-kDa bands using, as standards, different amounts of MBP–XPC probes purified by Ni^2+^ and heparin chromatography, as described before, and loaded in parallel onto the same gel.

### Electrophoretic mobility shift assays.

The synthetic 65-mer oligonucleotides 5′-CGGGGCGAATTCGAGCTCGCCCGGGATCCTCACATAGAGTCGACCTGCTGCAGCCCAAGCTTGGC-3′ and 5′-GCCAAGCTTGGGCTGCAGCAGGTCGACTCTATGTGAG GATCCCGGGCGAGCTCGAATTCGCCCCG-3′ were purchased from Microsynth. A DNA homoduplex was constructed by hybridizing these complementary oligonucleotides in 50 mM Tris-HCl, (pH 7.4), 10 mM MgCl_2_, and 1 mM dithiothreitol. The annealing was performed by heating to 95 °C for 10 min, followed by slow cooling (3 h at 25 °C). Electrophoretic mobility shift assays (reactions of 10 μl) were performed by incubating, at 20 °C for 30 min, ^32^P-labeled oligonucleotide substrate (2 nM), duplex poly[dI–dC] competitor DNA (10 ng/μl), and the indicated concentrations of XPC protein in 40 mM Tris-HCl, (pH 7.5), 5 mM MgCl_2_, 100 μg/ml bovine serum albumin, and 1 mM dithiothreitol [40]. Following the addition of gel loading buffer (2 μl) containing 30% (v/v) glycerol, 0.25% (w/v) bromophenol blue, and 0.25% (w/v) xylene cyanol in water, the extent of binding was determined on 7% native polyacrylamide gels.

### Screening of mutants for DNA binding.

Lysates (5 μl) from baculovirus-infected *Sf*9 cells [28] were mixed with 50 μl of single-stranded DNA agarose beads (Amersham) and 100 μl 25 mM Tris-HCl, (pH 7.5), 10% glycerol, 0.01% Triton X-100, 0.25 mM phenylmethane sulfonyl fluoride, 1 mM EDTA, supplemented by the indicated concentrations of NaCl. After incubation at 4 °C for 2 h, the supernatant was recovered and the beads were washed four times with 300 μl binding buffer. Finally, the DNA-bound proteins were eluted from the beads with 100 μl of 10 mM Tris-HCl, (pH 8.0), 1 mM EDTA, and 1% (w/v) sodium dodecylsulfate. Equivalent amounts of supernatant and DNA-bound fractions were loaded onto denaturing polyacrylamide gels, followed by immunoblot analysis, visualization by chemoluminescence (SuperSignal, Pierce, http://www.piercenet.com), and quantification by laser scanning densitometry.

The binding of mutants to single-stranded or double-stranded oligonucleotides was tested using purified MBP–XPC fusions obtained by immunoprecipitation. Paramagnetic beads (0.2 mg) containing the indicated amounts of wild-type or mutant XPC (between 10 and 100 ng) were incubated with ^32^P-labeled 65-mer probes (2 nM) in 200 μl of 25 mM Tris-HCl, (pH 7.5), 0.3 M NaCl, 10% glycerol, 0.01% Triton X-100, 0.25 mM phenylmethane sulfonyl fluoride, and 1 mM EDTA. Following an incubation of 90 min at 4 °C, the paramagnetic beads were washed three times with 200-μl binding buffer. Finally, the radiolabeled oligonucleotides associated with XPC protein were quantified by liquid scintillation counting. The background radioactivity resulting from unspecific binding of the oligonucleotides to empty beads (0.2 mg) was determined in separate reactions.

## Supporting Information

Table S1 Forward and Reverse Primers Used for Site-Directed Mutagenesis The mutated nucleotides are underlined.
(62 KB PDF)

## Abbreviations

GFP: green-fluorescent protein
GGR: global genome repair
MBP: maltose-binding protein
OB-fold: oligonucleotide/oligosaccharide-binding fold
RPA: replication protein A
SD: standard deviation
*Sf9,*: Spodoptera frugiperda
UV: ultraviolet
XP: xeroderma pigmentosum
XPC: xeroderma pigmentosum group C
