# Supplementary material for: An Aromatic Sensor with Aversion to Damaged Strands Confers Versatility to DNA Repair
Source: PLoS Biol. 2007 Mar 13;5(4):e79. doi: 10.1371/journal.pbio.0050079 (PMC1820611; doi:10.1371/journal.pbio.0050079)
Supplement: Supplementary file 1 [file pbio.0050079.st001.pdf]

Table 1

Forward and reverse primers used for site-directed mutagenesis. The mutated nucleotides are underlined.

Substitution	Oligonucleotide primer sequences
**W531G**	5’-GCTGGTATAGACCAG**G**GGCTAGAGGTGTTC-3’<br>5’-GAACACCTCTAGCC**C**CTGGTCTATACCAGC-3’
**W531A**	5’-GCTGGTATAGACCAGG**C**GCTAGAGGTGTTC-3’<br>5’-GAACACCTCTAGC**G**CCTGGTCTATACCAGC-3’
**W531S**	5’-GCTGGTATAGACCAGT**C**GCTAGAGGTGTTC-3’<br>5’-GAACACCTCTAGC**G**ACTGGTCTATACCAGC-3’
**F535A**	5’-CAGTGGCTAGAGGTG**GC**CTGTGAGCAGGAG-3’<br>5’-CTCCTGCTCACAG**GC**CACCTCTAGCCACTG-3’
**W542G**	5’-GAGCAGGAGGAAAAG**G**GGGTATGTGTAGAC-3’<br>5’-CTCCTCTGTCTGCC**C**CTTTTCCTCCTGCTC-3’
**W542A**	5’-GAGCAGGAGGAAAAGG**C**GGTATGTGTAGAC-3’<br>5’-CTCCTCTGTCTGCG**C**CTTTTCCTCCTGCTC-3’
**W542S**	5’-GAGCAGGAGGAAAAGT**C**GGTATGTGTAGAC-3’<br>5’-CTCCTCTGTCTGC**G**ACTTTTCCTCCTGCTC-3’
**Y585A**	5’-GATGTCACACAGAGG**GC**CGACCCAGTCTGG-3’<br>5’-CCAGACTGGGTCG**GC**CCTCTGTGTGACATC-3’
**Y585G**	5’-GATGTCACACAGAGGG**G**CGACCCAGTCTGG-3’<br>5’-CCAGACTGGGTCG**C**CCCTCTGTGTGACATC-3’
**Y585S**	5’-GATGTCACACAGAGG**T**CCGACCCAGTCTGG-3’<br>5’-CCAGACTGGGTCGG**A**CCTCTGTGTGACATC-3’
**K595G**	5’-GGATGACAGTGACCCGC**GG**GTGCCGGGTTGATGCTG-3’<br>5’-CAGCATCAACCCGGCAC**CC**GCGGGTCACTGTCATCC-3’
**R597G**	5’-CAGTGACCCGCAAGTGC**G**GGGTTGATGCTGAGTGG-3’<br>5’-CCACTCAGCATCAACCC**C**GCACTTGCGGGTCACTG-3’
**P635A**	5’-CACATGGACCAGCCTTTG**G**CCACTGCCATTGGC-3’<br>5’-GCCAATGGCAGTGG**C**CAAAGGCTGGTCCATGTG-3’
**Y641A**	5’-CCCACTGCCATTGGCTTA**GC**TAAGAACCACCCTCTG-3’<br>5’-CAGAGGGTGGTTCTTA**GC**TAAGCCAATGGCAGTGGG-3’
**K642G**	5’-CTGCCATTGGCTTATAT**GG**GAACCACCCTCTGTATGC-3’<br>5’-GCATACAGAGGGTGGTTC**CC**ATATAAGCCAATGGCAG-3’
**H644A**	5’-GGCTTATATAAGAAC**GC**CCCTCTGTATGCCCTG-3’<br>5’-CAGGGCATACAGAGGG**GC**GTTCTTATATAAGCC-3’
**Y647A**	5’-GAACCACCCTCTG**GC**TGCCCTGAAGCGGCATCTC-3’<br>5’-GAGATGCCGCTTCAGGGCA**GC**CAGAGGGTGGTTC-3’
**R651G**	5’-CCTCTGTATGCCCTGAAG**G**GGCATCTCCTGAAATATG-3’<br>5’-CATATTTCAGGAGATGCC**C**CTTCAGGGCATACAGAGG-3’

**K655G**	5'-GAAGCGGCATCTCCTG<u>**GG**</u>ATATGAGGCCATCTATCC-3'<br>5'-GGATAGATGGCCTCATAT<u>**CC**</u>CAGGAGATGCCGCTTC-3'
**Y656A**	5'-CGGCATCTCCTGAAA<u>**GC**</u>TGAGGCCATCTATCCC-3'<br>5'-GGGATAGATGGCCTCA<u>**GC**</u>TTTCAGGAGATGCCG-3'
**Y660A**	5'-GAAATATGAGGCCATC<u>**GC**</u>TCCCGAGACAGCTGCC-3'<br>5'-GGCAGCTGTCTCGGGA<u>**GC**</u>GATGGCCTCATATTTC-3'
**Y676A**	5'-CGTGGAGAAGCGGTC<u>**GC**</u>CTCCAGGGATTGTGTG-3'<br>5'-CACACAATCCCTGGAG<u>**GC**</u>GACCGCTTCTCCACG-3'
**R678G**	5'-GAAGCGGTCTACTCC<u>**G**</u>GGGATTGTGTGCACAC-3'<br>5'-GTGTGCACACAATCCC<u>**C**</u>GGAGTAGACCGCTTC-3'
**S686A**	5'-GTGCACACTCTGCAT<u>**G**</u>CCAGGGACACGTGGCTG-3'<br>5'-CAGCCACGTGTCCCTGG<u>**C**</u>ATGCAGAGTGTGCAC-3'
**W690G**	5'-GCATTCCAGGGACACG<u>**G**</u>GGCTGAAGAAAGCAAGAG-3'<br>5'-CTCTTGCTTTCTTCAGCC<u>**C**</u>CGTGTCCCTGGAATGC-3'
**W690A**	5'-GCATTCCAGGGACACG<u>**G**</u>CGCTGAAGAAAGCAAGAG-3'<br>5'-CTCTTGCTTTCTTCAGCG<u>**C**</u>CGTGTCCCTGGAATGC-3'
**W690S**	5'-GCATTCCAGGGACACGT<u>**C**</u>GCTGAAGAAAGCAAGAG-3'<br>5'-CTCTTGCTTTCTTCAGC<u>**G**</u>ACGTGTCCCTGGAATGC-3'
**K693G**	5'-GGACACGTGGCTGAAG<u>**GG**</u>AGCAAGAGTGGTGAGG-3'<br>5'-CCTCACCACTCTTGCT<u>**CC**</u>CTTCAGCCACGTGTCC-3'
**R698G**	5'-GAAAGCAAGAGTGGTG<u>**G**</u>GGCTTGGAGAAGTACC-3'<br>5'-GGTACTTCTCCAAGCC<u>**C**</u>CACCACTCTTGCTTTC-3'
**Y704A**	5'-CTTGGAGAAGTACCC<u>**GC**</u>CAAGATGGTGAAAGGC-3'<br>5'-GCCTTTCACCATCTTG<u>**GC**</u>GGGTACTTCTCCAAG-3'
**K705G**	5'-GCTTGGAGAAGTACCCTAC<u>**GG**</u>GATGGTGAAAGGCTTTTC-3'<br>5'-GAAAAGCCTTTCACCATC<u>**CC**</u>GTAGGGTACTTCTCCAAGC-3'
**K708G**	5'-CCCTACAAGATGGTG<u>**GG**</u>AGGCTTTTCTAACCGTGC-3'<br>5'-GCACGGTTAGAAAAGCCT<u>**CC**</u>CACCATCTTGTAGGG-3'
**F733A**	5'-GAAAATGACCTGGGCCTG<u>**GC**</u>TGGCTACTGGCAGACAGAG-3'<br>5'-CTCTGTCTGCCAGTAGCCA<u>**GC**</u>CAGGCCCAGGTCATTTTC-3'
**F733G**	5'-GAAAATGACCTGGGCCTGG<u>**G**</u>TGGCTACTGGCAGACAGAG-3'<br>5'-CTCTGTCTGCCAGTAGCCA<u>**C**</u>CCAGGCCCAGGTCATTTTC-3'
**F733S**	5'-GAAAATGACCTGGGCCTG<u>**T**</u>CTGGCTACTGGCAGACAGAG-3'<br>5'-CTCTGTCTGCCAGTAGCCAG<u>**A**</u>CAGGCCCAGGTCATTTTC-3'
**W736G ?**	5'-GGCCTGTTTGGCTAC<u>**G**</u>GGCAGACAGAGGAG-3'<br>5'-CTCCTCTGTCTGCC<u>**C**</u>GTAGCCAAACAGGCC-3'
**Y741A**	5'-GGCAGACAGAGGAG<u>**GC**</u>TCAGCCCCCAGTG-3'<br>5'-CACTGGGGGCTGA<u>**GC**</u>CTCCTCTGTCTGCC-3'
**F756A**	5'-GTGCCCCGGAACGAG<u>**GC**</u>TGGGAATGTGTAC-3'<br>5'-GTACACATTCCCA<u>**GC**</u>CTCGTTCCGGGGCAC-3'
**F762A**	5'-GGGAATGTGTACCTC<u>**GC**</u>CCTGCCCAGCATG-3'<br>5'-CATGCTGGGCAGG<u>**GC**</u>GAGGTACACATTCCC-3'
**F762G**	5'-GGGAATGTGTACCTCG<u>**G**</u>CCTGCCCAGCATG-3'<br>5'-CATGCTGGGCAGG<u>**C**</u>CGAGGTACACATTCCC-3'
**F762S**	5'-GGGAATGTGTACCTC<u>**T**</u>CCCTGCCCAGCATG-3'<br>5'-CATGCTGGGCAGGG<u>**A**</u>GAGGTACACATTCCC-3'

**F797A**	5’-CAGGCCATCACTGGC**GC**TGATTTCCATGGCGG-3’<br>5’-CCGCCATGGAAATCA**GC**GCCAGTGATGGCCTG-3’
**F799A**	5’-CCATCACTGGCTTTGAT**GC**CCATGGCGGCTACTC-3’<br>5’-GAGTAGCCGCCATGG**GC**ATCAAAGCCAGTGATGG-3’
